# Supplementary material for: Development of New PCR Assay with SYBR Green I for Detection of Mycoplasma, Acholeplasma, and Ureaplasma sp. in Cell Cultures
Source: Diagnostics (Basel). 2021 May 14;11(5):876. doi: 10.3390/diagnostics11050876 (PMC8156504; doi:10.3390/diagnostics11050876)
Supplement: Supplementary file 1 [file diagnostics-11-00876-s001.zip › diagnostics-1210943-SI.pdf]

Table S1. DNA standards used in this study.

DNA standards used for primer's specificity confirmation.		DNA standards used for primer's cross-reactivity evaluation.	
Species	NCTC No[1*]	Species	DNA
A. laidlawii	10116	*S. enteritidis*	ATCC[2*]
M. hyorhinis.	10130	*E. faecalis*	ATCC
M. fermentans	010117	*L. fermenti*	ATCC
M. hominis	10111	*C. albicans*	Clinical isolate after VITEK System identification
M. orale	010112	*C. tropicalis*	Clinical isolate after VITEK System identification
M. salivarium	10113	*S. carlsbergensis*	ATCC
M. arginini	10129	*H. sapiens*	GM14467 (B-lymphoid cells), CB[3*]
M. genitalium	10195	*M. musculus*	L929 (fibroblasts), ATCC
M. pneumoniae	010119		
U. urealyticum	10177		

[1*] National Collection of Type Cultures (NCTC), UK; [2*] American Type Culture Collection (ATCC); [3*]Coriell Bank (CB), USA.

Table S2. Description of primers used for selected *Mycoplasma, Acholeplasma* and *Ureaplasma* species detection arranged in Test-1.

Gene; Gene Bank Sequence No	Forward/ Reversed Primer Fw/Rv	Sequence of primer pairs 5′→3′ [1*]	Specific primer rate %	Primer melting temperature- Tm °C	GC contents %	Harpins ΔG kcal/mol	Dimers ΔG kcal/mol	PCR product Sizes bp
**M1 primers** specific for *A. laidlawii, M. arginini, M. hominis, M. orale, M. salivarium*								
16S RNA; 16S-23S ITS; 23S rRNA	M1 Fw	CCACATTGGGACTGAGA	70.5	62	52.9	-1.6	-1.6	201÷214
JN935890.1	M1 Rv	CTGCTGGCACATAGTTAG	80.2	61.3	50.0	0.0	-2.0	
**M2 primers** specific for *M. arginini, M. hominis, M. orale, M. salivarium, M. hyorhinis, M. fermentans*								
16S RNA;	M2 Fw	TTACCCACTCTTGACATC	81.7	59.7	44.4	0.0	0.0	227÷235
AB353273	M2 Rv	ACTCGTAAGAGGCATGAT	76.9	61.6	44.4	-1.0	-2.4	

**M3 primers** specific for *M. pneumoniae, M. pirum, M. genitalium* and *U. urealyticum*								
16S RNA;	M3 Fw	GGTAGTACATTCGCAAGA	85.8	60.2	44.4	0.0	2.1	209
AY466443	M3 Rv	GGACTTAACCCAACATCTC	75.4	61.4	47.4	0.0	-0.9	
**ACTB primers** specific for *ACTB gene* of *H. sapiens* - internal control								
ACTB	ACTB Fw	TCGTGCGTGACATTAAGG	64.9	64.2	50.0	0.0	-0.9	176
NM_001101	ACTB Rv	GAAGGAAGGCTGGAAGAGTG	65.3	64.3	55.0	0.0	0.0	

[1*] Primer sequences were designed using Beacon Designer v. 7.91 software (Pimer Biosoft, USA); ΔG – the Gibbs free energy was calculated according to the formula: ΔG=ΔH-TΔS; ΔH – enthalpy; ΔS – entropy. Optimally a 3′ end hairpin with a ΔG of -2 kcal/mol and an internal hairpin with a ΔG of -3 kcal/mol is generally tolerated. Optimally a 3′ end self-dimer with a ΔG of -5 kcal/mol and an internal self-dimer with a ΔG of -6 kcal/mol is generally tolerated. Specificity of M1, M2 and M3 primers was confirmed by BLAST alignment. No specificity for *H. sapiens. M. musculus. R. norvegicus*.

Table S3. Description of primers used for bacterial species identification from *Mycoplasma*/*Acholeplasma*/*Ureaplasma* sp. and for internal control.

Species	Sequence No / Gene symbol	Forward/ Reversed Primer Fw/Rv	Sequence of primer pairs 5′→3′ [1*]	Specific primer rate %	Primer melting temperature °C	GC contents %	Harpins ΔG	Dimers ΔG	PCR product size bp
A. laidlawii	5804530	Fw	AAATAATTTTGAGGATGCG	64.0	55.5	31.6	-0.2	-1.1	143
	trmA	Rv	ATGAACCATAGACTAATCG	88.9	55.3	36.8	-0.6	-0.6	
M. hyorhinis	9712706	Fw	TCCAGTAAATGTCTTGATT	81.1	55.2	31.6	0.0	0.0	108
	eut	Rv	ATGCCTGTAATAATTGGT	85.3	54.8	33.3	0.0	-1.1	
M. fermentans	10113402	Fw	ACTTCGTTACAAGAAATTAC	78.2	57.4	30.0	-0.6	-1.2	119
	dnaA	Rv	AACAACAGCAACAGTATA	85.9	57.5	33.3	0.0	-0.3	
M. hominis	8595493	Fw	CCAACTATAATCAAGGCATT	88.1	57.1	35.0	0.0	-0.3	190
	acpD/ACP	Rv	TTCACATTCCTTCAAGATAA	89.9	57.9	30.0	0.0	-0.6	
M. orale	FJ888600.1	Fw	ATCCCTAAAGGTGAAGAA	79.8	58.5	38.9	0.0	-1.4	174
	rpoB	Rv	ACGGCTAATAATTTCTACG	76.7	58.6	36.8	0.0	-1.2	
M. salivarium	FJ765340	Fw	GCTGGTGAAGATATTCTAA	88.9	57.8	36.8	-0.8	-0.8	142
	rpoB	Rv	CACCTTTAGGAGAAGTAC	76.6	57.3	44.4	-0.6	-2.1	
M. arginini	DQ272351.1	Fw	TGCTCAATCAACTGTAAA	81.9	57.8	33.3	0.0	0.0	112
	rpoB	Rv	CCCAACAAAATCAACTTC	73.5	57.9	38.9	0.0	0.0	
M. genitalium	875315	Fw	CTCAATTCAACAAGTTAAACC	78.7	57.3	33.3	0.0	-1.1	110

	ftsZ	Rv	ATTAGGATAGTGATAAAGTGAT G	81.1	57.3	30.4	0.0	0.0	
M. pneumoniae	876854	Fw	CACCTTCTTCACTAACAA	90.6	55.4	38.9	0.0	0.0	134
	gap	Rv	GGACAACACCTTAATGAT	89.1	55.5	38.9	0.0	-0.9	
M. pirum	DQ514614	Fw	CGTTCTTCACAAGAGTTA	84.0	57.9	38.9	-1.9	-1.9	116
	rpoB	Rv	GCAGAGTCATCATTTTCTA	72.0	58.4	36.8	-0.8	-0.8	
U. urealyticum	6984329	Fw	GGAACTGGAGGACATAAC	90.6	58.1	50.0	0.0	0.0	175
	dcm	Rv	TTACGACAGGAACTGATAC	85.3	57.7	42.1	-1.2	-1.2	
H. sapiens	NM_001101	Fw	TCGTGCGTGACATTAAGGAG	64.9	64.2	50.0	0.0	-0.9	176
	ACTB [2*]	Rv	GAAGGAAGGCTGGAAGAGTG	65.3	64.3	55.0	0.0	0.0	

[2*] ACTB – specific primers for beta-actin gene of *H. sapiens, M. musculus* 4r54 used in this study for internal control (IC) detection in DNA originated from human or mouse cell cultures. All primers for species detection characterized no specificity for *H. sapiens. M. musculus. R. norvegicus* and all bacterial genomes by BLAST alignment.

Table S4. Precision evaluation results obtained for Test-1 performed with ACTB. Within-run precision (RSDw) was calculated for each of independent experiment and expressed by coefficients of variation (%CV) for and between-run precision (RSDb) was calculated for three independent experiments.

Rep-lica-te	I experiment						II experiment					III experiment					I-III experiments		
	GM14467 DNA (ng/µl)	Ct (dRn)	Tm*	Ave-rage Ct	SD Ct	RSDw CV% **	Ct (dRn)	Tm	Ave-rage Ct	SD Ct	RSDw CV%	Ct (dRn)	Tm	Ave-rage Ct	SD Ct	RSDw CV%	Ave-rage Ct	SD Ct	RSDb CV%
1	1.25x10^{3}	12.27	87.28	12.22	0.06	0.50	12.94	87.28	12.88	0.07	0.52	12.94	87.05	13.21	0.27	2.04	12.77	0.52	4.11
1	1.25x10^{3}	12.23	87.28				12.90	87.28				13.48	87.05						
1	1.25x10^{3}	12.15	87.28				12.81	87.28				13.21	87.05						
2	6.25x10^{-1}	13.15	87.28	13.24	0.12	0.89	13.97	86.75	13.95	0.15	1.08	13.70	87.10	14.05	0.35	2.49	13.74	0.43	3.13
2	6.25x10^{-1}	13.37	87.28				13.79	86.75				14.39	87.00						
2	6.25x10^{-1}	13.19	86.75				14.09	86.75				14.05	87.05						
3	3.12x10^{-1}	13.94	87.25	13.96	0.03	0.25	14.87	86.72	14.84	0.06	0.41	15.24	86.95	14.91	0.33	2.21	14.57	0.49	3.35
3	3.12x10^{-1}	14.00	86.72				14.88	86.72				14.57	87.10						
3	3.12x10^{-1}	13.94	86.72				14.77	86.72				14.91	87.05						
4	1.56x10^{-1}	14.92	87.22	14.92	0.05	0.30	15.90	86.68	15.80	0.11	0.71	16.77	87.40	17.01	0.23	1.3	15.91	0.91	5.75
4	1.56x10^{-1}	14.97	86.68				15.68	86.68				17.24	86.95						

4	1.56x10^{-1}	14.88	86.68				15.83	86.68				17.01	87.05						
5	7.81x10^{-2}	15.90	87.22	15.86	0.09	0.55	17.13	87.22	16.99	0.17	1.00	18.87	87.40	19.01	0.14	0.7	17.28	1.38	8.01
5	7.81x10^{-2}	15.76	87.22				17.03	86.68				19.14	87.40						
5	7.81x10^{-2}	15.92	86.68				16.80	86.68				19.01	87.05						

*Tm-Product (-R'(T)); CV%** - Coefficient of variation (CV) – RSD x 100%; RSD = S/X; S-Standard deviation; X – mean value (average).

Table S5. Precision results obtained for Test-1 performed with M1, M2, M3 primers and expressed as relative repeatability standard deviation (RSD) within-laboratory variations a) RSD intra-assays[1*]; b) RSD inter-assays[2*].

-

a)	Precision for M1 primers with *M. orale* standard n=6				Precision for M2 primers with *M. orale* DNA standard n=6				Precision for M3 primers with *U. urealyticum* DNA standard n=6		
DNA (cn/μl)	Ave-rage Ct(dRn)	SD Ct	RSDw[1*] CV%	DNA (cn/μl)	Ave-rage Ct(dRn)	SD Ct	RSDw[1*] CV%	DNA (cn/μl)	Ave-rage Ct(dRn)	SD Ct	RSDw[1*] CV%
2.5x10^{5}	22.31	0.43	1.91	1.8x10^{5}	22.52	0.67	2.95	5.0x10^{2}	27.06	0.57	2.12
2.5x10^{4}	26.19	0.41	1.57	5.1x10^{3}	28.04	0.40	1.43	5.0x10^{1}	30.74	0.28	0.90
2.5x10^{3}	30.05	0.60	1.98	7.2x10^{1}	33.21	0.49	1.48	5.0x10^{0}	33.20	0.22	0.65
2.5x10^{2}	34.94	0.47	1.34	5.0x10^{0}	39.18	0.28	0.70	2.5x10^{-1}	39.12	0.49	1.27
2.5x10^{0}	39.21	0.50	1.28								

b)	Precision for M1 primers with *M. salivarium* DNA standard n=6			Precision for M2 primers with *M. fermentans* DNA standard n=9			Precision for M3 primers with *M. pneumoniae* DNA standard n=10		
DNA (cn/μl)	Ave-rage Ct(dRn)	SD Ct	RSDw[2*] CV%	Ave-rage Ct(dRn)	SD Ct	RSDw[2*] CV%	Ave-rage Ct(dRn)	SD Ct	RSDw[2*] CV%
2.5x10^{4}	26.80	1.54	5.75	27.09	1.49	5.49	27.34	1.82	6.69

Table S6. Robustness evaluation results obtained for Test-1 performed with M1, M2, M3 and ACTB primers, different template standards and three types of the plastic tubes in appropriate experiments (E) I-III*. Each experiment was performed in duplicates.

Species	Primer	EI	RSD1	EII	RSD2	EIII	RSD3	EI-III	RSD1-3

of DNA standard	pair	Ct average	SD	(%)	Ct average	SD	(%)	Ct average	SD	(%)	Ct average	SD	(%)
A. laidlawii	M1	21.09	0.11	0.54	26.58	0.89	3.34	28.32	1.49	5.26	25.33	3.77	14.90
M. arginini		24.74	0.66	2.66	ND**	ND	ND	31.01	2.57	8.28	27.87	4.43	15.91
M. hominis		22.66	0.83	3.65	25.81	1.09	4.23	ND	ND	ND	24.23	2.23	9.20
M. orale		25.65	0.19	0.74	ND	ND	ND	32.31	1.60	4.96	28.98	4.71	16.26
M. fermentans	M2	23.70	2.03	8.55	25.65	0.95	3.71	28.00	0.67	2.38	25.78	2.15	8.36
M. hyorhinis		25.89	0.19	0.74	30.15	2.62	8.69	33.78	0.13	0.40	29.94	3.95	13.19
M. orale		25.44	1.14	4.48	ND	ND	ND	34.86	1.35	3.87	30.15	6.66	22.10
U. urealyticum	M3	22.73	0.90	3.97	24.41	0.64	2.62	27.30	0.72	2.64	24.81	2.31	9.33
M. pneumoniae		26.08	0.94	3.60	29.50	0.64	2.16	ND	ND	ND	27.79	2.42	8.70
M. genitalium		23.67	0.62	2.63	25.54	0.07	0.26	27.30	0.42	1.53	25.50	1.81	7.11
H. sapiens	ACTB	17.32	0.40	2.29	18.80	0.96	5.09	19.76	0.52	2.65	18.62	1.23	6.62

* EI: tube strips with separate cap strips; Eppendorf, Germany; EII: tube strips with separate cap strips; Googlab Scientific, USA ; EIII: separate tubs with caps Googlab Scientific, USA; **ND - No data

M1

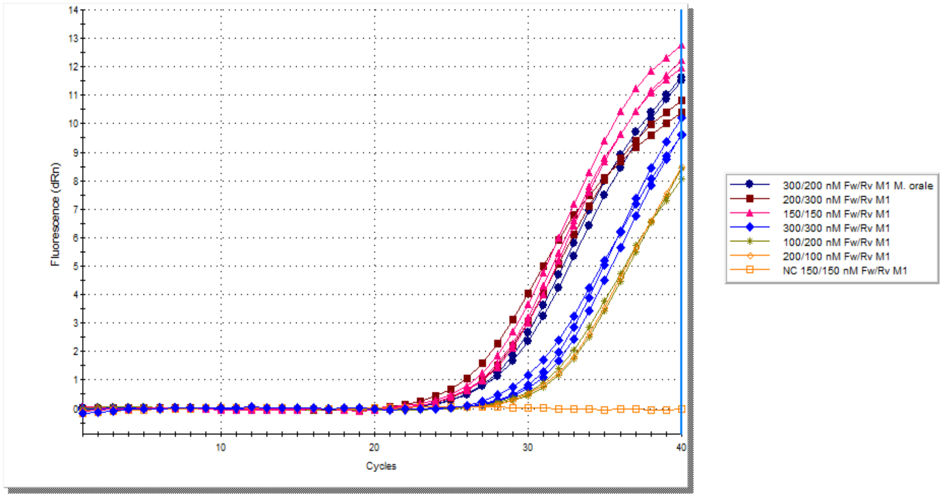
Fluorescence (dRn)
Cycles
300/200 nM Fw/Rv M1 M. orale
200/300 nM Fw/Rv M1
150/150 nM Fw/Rv M1
300/300 nM Fw/Rv M1
100/200 nM Fw/Rv M1
200/100 nM Fw/Rv M1
NC 150/150 nM Fw/Rv M1

M1

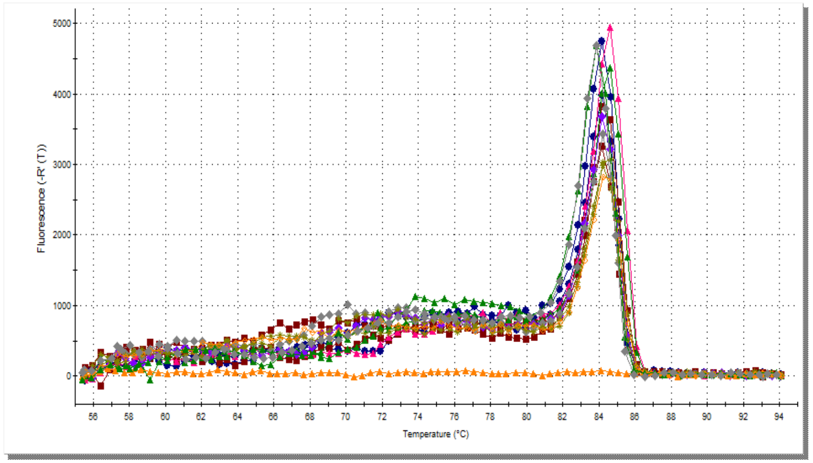
Fluorescence (-R' (T))
Temperature (°C)

M2

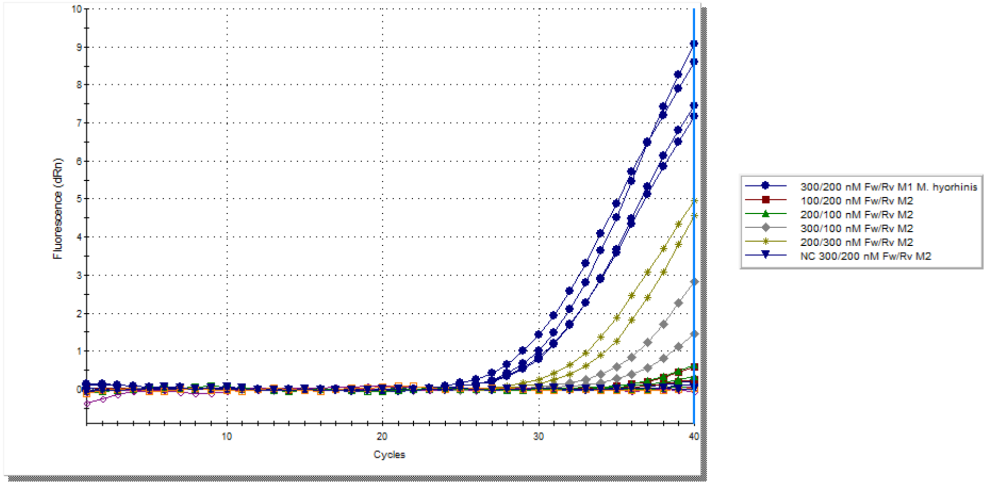
Fluorescence (dRn)
Cycles
300/200 nM Fw/Rv M1 M. hyorhinis
100/200 nM Fw/Rv M2
200/100 nM Fw/Rv M2
300/100 nM Fw/Rv M2
200/300 nM Fw/Rv M2
NC 300/200 nM Fw/Rv M2

M2

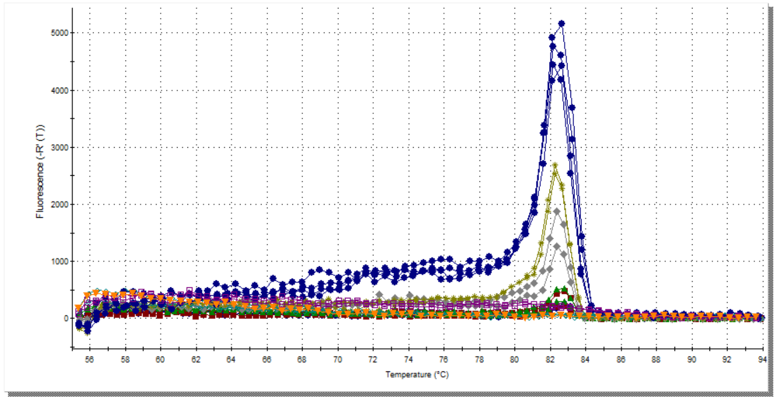
Fluorescence (-R' (T))
Temperature (°C)

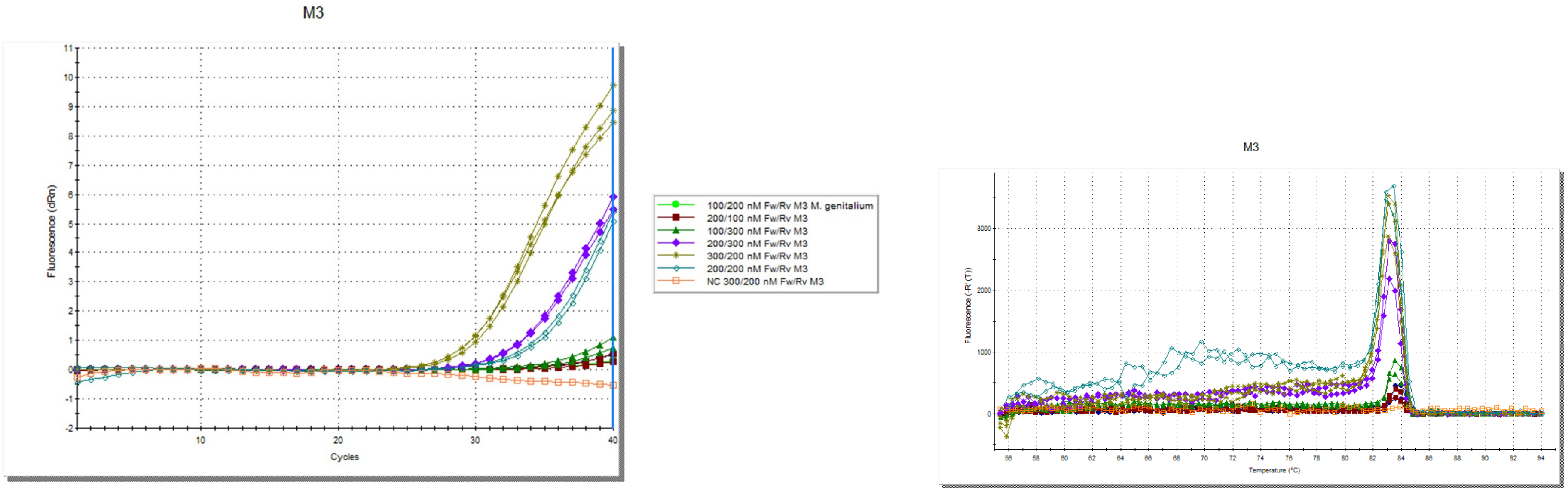

Figure S1. M1, M2, and M3 forward (Fw), reversed (Rv) primers optimization.

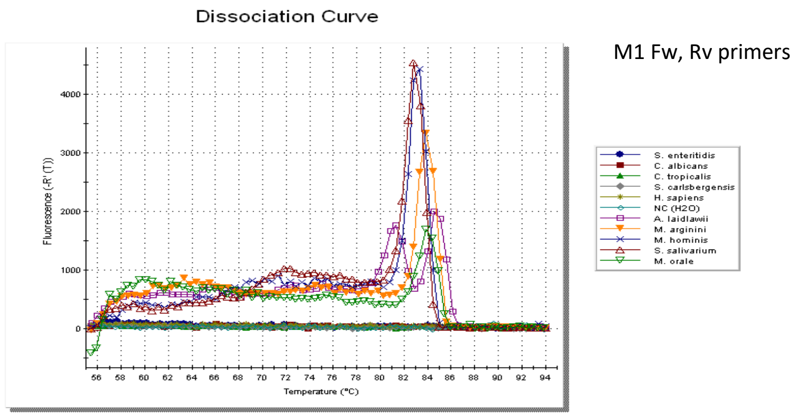

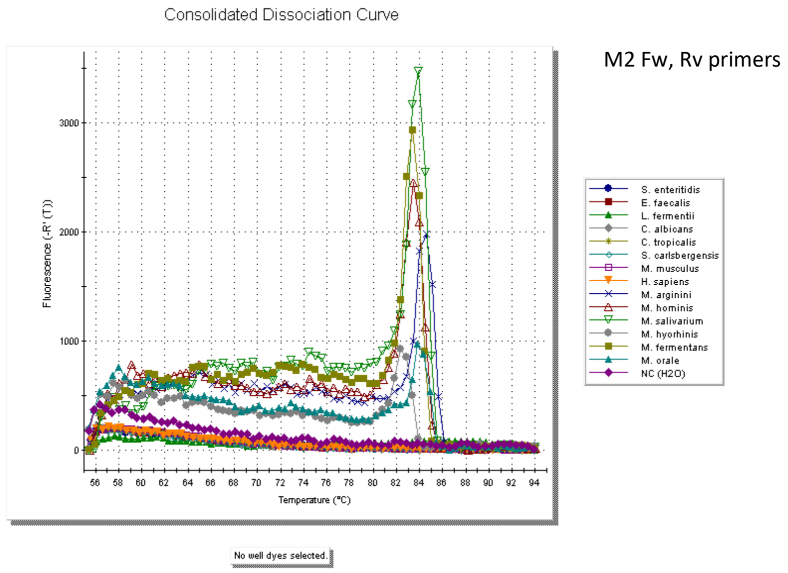

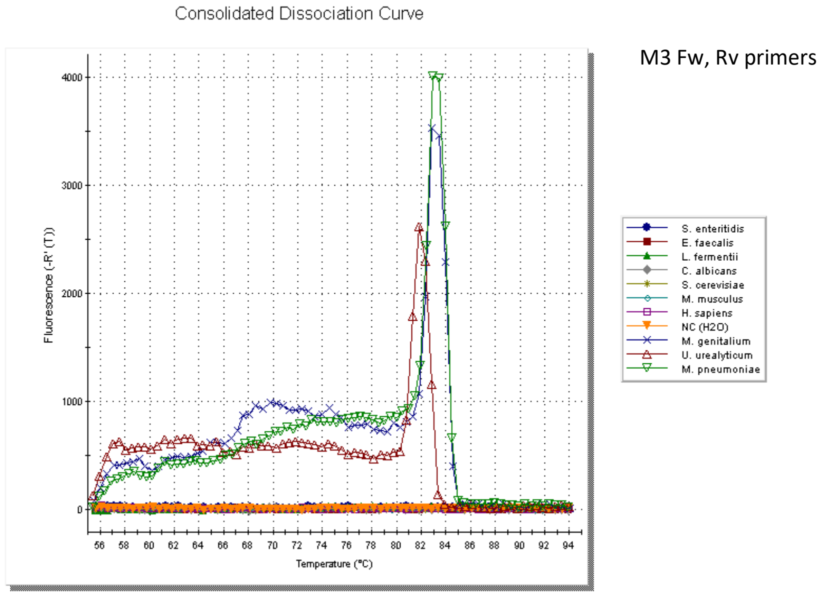

Figure S2. Analysis of the specificity of qPCRs products found in Test-1 v.2. Dissociation curves obtained for M1, M2 and M3 forward (Fw) and reversed (Rv) primers and 0.05 ng of DNA standards from *Mycoplasma*, *Acholeplasma* and *Ureaplasma* subgroup -

1, -2, -3 respectively. No qPCR products were found with all tested primers and nonrelated DNA standards (*S. enteritidis, C. albicans, C. tropicalis, S. carlsbergensis, H. sapiens* and *M. musculus*); NC - negative controls.

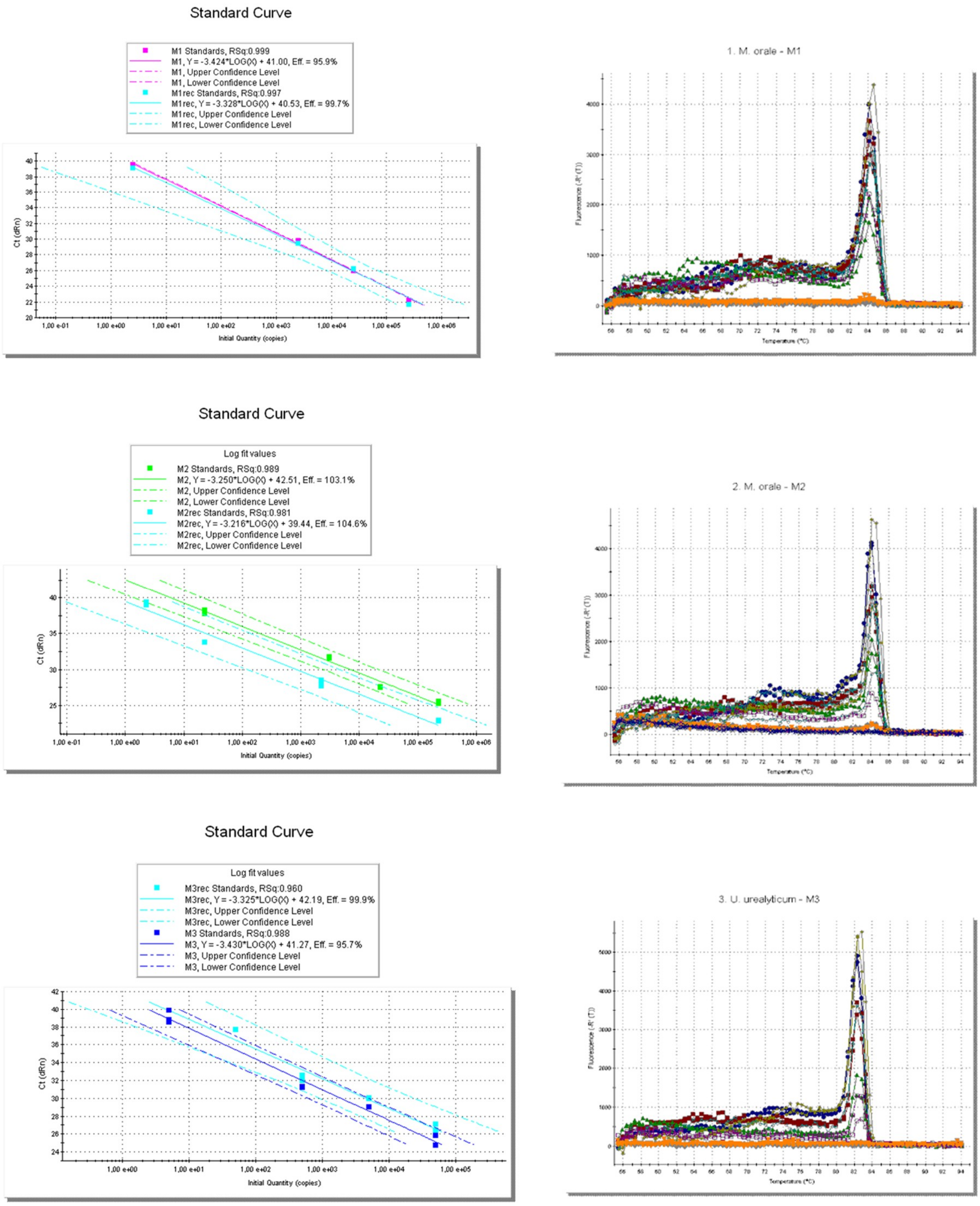

Figure S3. Analysis results obtained for human DNA samples spiked with *M. orale* DNA standard in Test-1 v.2 performed with M1 and M2 primers and *U. urealyticum* DNA standard with M3 primers a) efficiency of qPCR in recovery (rec) evaluation; b) specificity of amplicons obtained in Dissociation curves of spiked human DNA (from GM14467 cells) by *Mycoplasma* templates.

1. M. hyorhinis

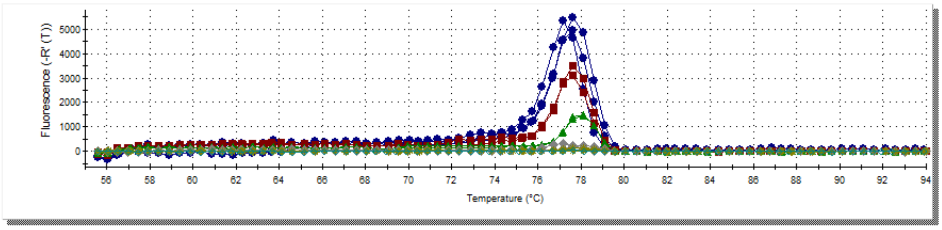

2. M. fermentans

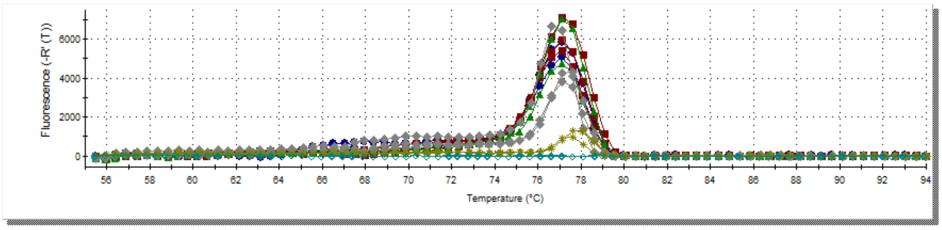

3. M. hominis

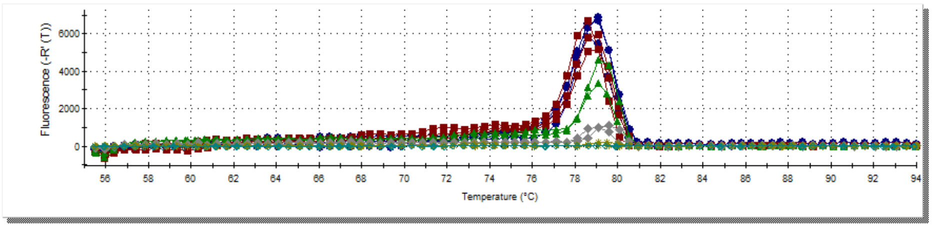

4. M. orale

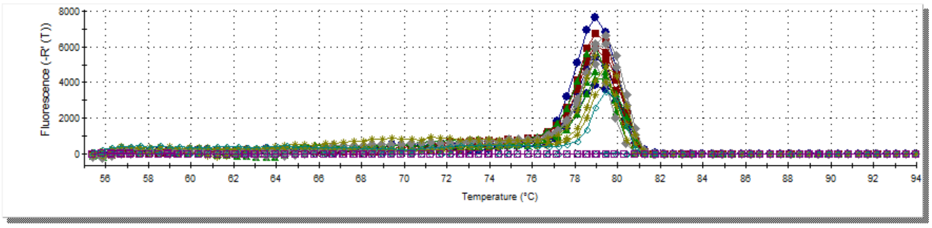

5. M. salivarium

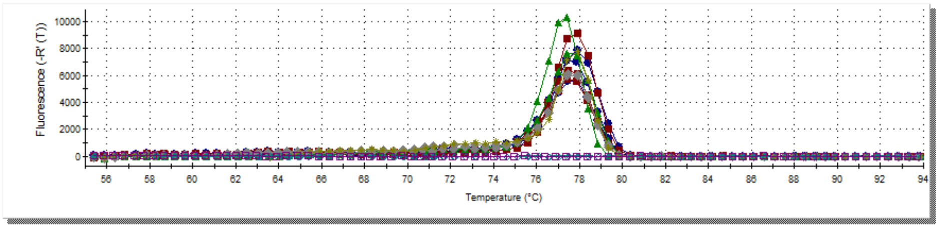

6. M. arginini

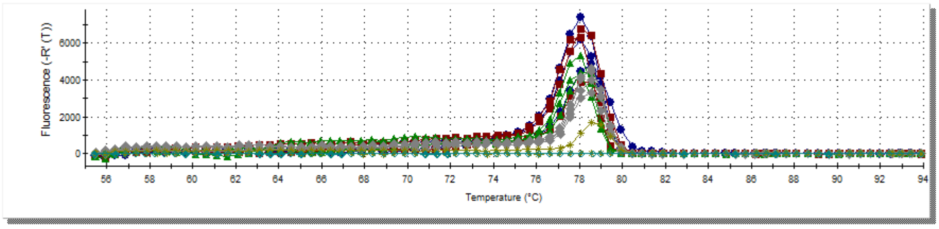

7. M. genitalium

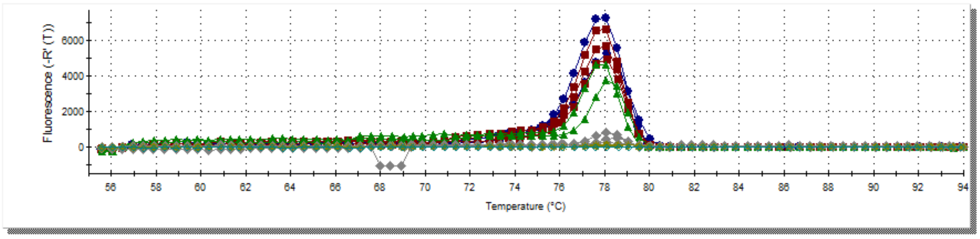

8. M. pneumoniae

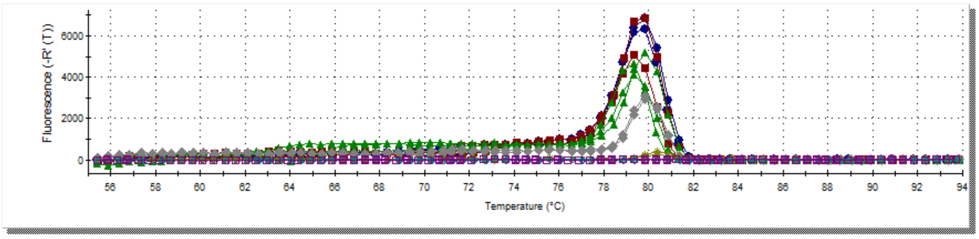

9. U. urealyticum

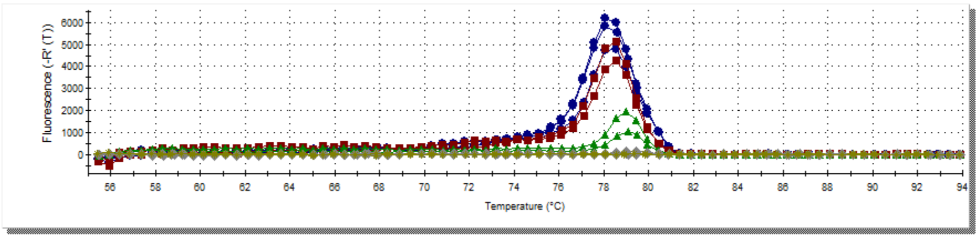

10. A. laidlawii

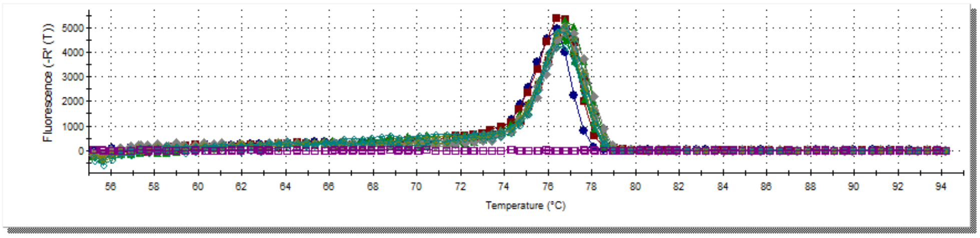

Figure S4. The specificity of qPCRs products obtained in Test-2 includes melting curves with maximum temperature values specific for primer pairs and DNA template dilutions in a range showed in Table 2.
